# Hand and Grasp Selection in a Preferential Reaching Task: The Effects of Object Location, Orientation, and Task Intention

**DOI:** 10.3389/fpsyg.2016.00360

**Published:** 2016-03-16

**Authors:** Sara M. Scharoun, Kelly A. Scanlan, Pamela J. Bryden

**Affiliations:** ^1^Department of Kinesiology, University of Waterloo, WaterlooON, Canada; ^2^Department of Kinesiology and Physical Education, Wilfrid Laurier University, WaterlooON, Canada

**Keywords:** preferential reaching, hand selection, grasp selection, object location, object orientation, task intention

## Abstract

As numerous movement options are available in reaching and grasping, of particular interest are what factors influence an individual’s choice of action. In the current study a preferential reaching task was used to assess the propensity for right handers to select their preferred hand and grasp a coffee mug by the handle in both independent and joint action object manipulation contexts. Mug location (right-space, midline, and left-space) and handle orientation (toward, away, to left, and to right of the participant) varied in four tasks that differed as a function of intention: (1) pick-up (unimanual, independent); (2) pick-up and pour (bimanual, independent); (3) pick-up and pass (unimanual, joint action); and (4) pick-up, pour and pass (bimanual, joint action). In line with previous reports, a right-hand preference for unimanual tasks was observed. Furthermore, extending existing literature to a preferential reaching task, role differentiation between the hands in bimanual tasks (i.e., preferred hand mobilizing, non-preferred hand stabilizing) was displayed. Finally, right-hand selection was greatest in right space, albeit lower in bimanual tasks compared to what is typically reported in unimanual tasks. Findings are attributed to the desire to maximize biomechanical efficiency in reaching. Grasp postures were also observed to reflect consideration of efficiency. More specifically, within independent object manipulation (pick-up; pick-up and pour) participants only grasped the mug by the handle when it afforded a comfortable posture. Furthermore, in joint action (pick-up and pass; pick-up, pour and pass), the confederate was only offered the handle if the intended action of the confederate was similar or required less effort than that of the participant. Together, findings from the current study add to our knowledge of hand and grasp selection in unimanual and bimanual object manipulation, within the context of both independent and joint action tasks.

## Introduction

As there are an almost infinite number of options available to complete any given movement, of particular interest are what factors influence an individual’s choice of action. When reaching for objects, hand preference is consistently observed to influence hand selection. Preferred hand use is displayed at the midline and in ipsilateral space, and right-handers are more likely to adopt this pattern of hand selection when reaching for objects in contralateral space (Bryden et al., 2000, 2011; Gabbard and Rabb, 2000; Bryden and Roy, 2006; Mamolo et al., 2006; Bryden and Huszczynski, 2011). The *motor dominance hypothesis* argues that if preference for one hand truly exists, then that hand will be selected to perform most unimanual tasks (Bryden et al., 1994). However, motor dominance is not the only hypothesis used to explain hand selection. According to the *kinaesthetic hypothesis*, biomechanical constraints should decrease preferred hand use in contralateral space to limit awkward postures; therefore, hand selection will be constrained by object proximity and efficiency of the movement. Furthermore, the *hemispheric bias hypothesis* indicates that each hand typically performs best in its own region of space due to spatial compatibility (Gabbard and Rabb, 2000).

To differentiate between the three aforementioned hypotheses, Gabbard and Helbig (2004) modified a traditional preferential reaching paradigm with a crossed arms condition. Right-handed participants reached for small cubes located at seven locations in hemispace, starting with arms uncrossed, and subsequently crossed. Typical outcomes (i.e., preferred hand use in ipsilateral space and at midline, etc.) were demonstrated in uncrossed conditions; whereas, hand selection in the crossed condition was driven by object proximity. Results provided support for the *kinaesthesis* hypothesis (Gabbard and Helbig, 2004). Nevertheless, interpretation was limited, as the manner in which hand were crossed did not vary (i.e., right crossed over left hand).

Bryden and Huszczynski (2011) extended Gabbard and Helbig’s (2004) study to include both crossed-arms conditions (i.e., right crossed over left hand, left crossed over right hand). In addition, biomechanical constraints were assessed by manipulating the handle orientation of a mug in relation to the participant’s hands, where participants were required to grasp the handle. Finally, two tasks (pick-up, and pick-up-and-use) used by Bryden et al. (2003) were included to assess the role of task complexity. Unlike Gabbard and Helbig (2004), Bryden and Huszczynski (2011) observed that object proximity did not play a significant role in hand selection as participants typically selected the hand on top in the cross arm condition for use. Instead, variance in reaching was attributed to handle orientation, or rather, the biomechanical constraints imposed when reaching for the handle. Findings also revealed that task complexity was not influential. This was attributed to the complex task being limited to pantomime (i.e., pretending to drink); therefore, an end goal was not clearly defined (Bryden and Huszczynski, 2011).

Although tools have been shown to elicit greater preferred-hand selection compared to ecologically irrelevant objects (e.g., dowel), performing the action afforded by the tool leads to a further increase in preferred-hand selection compared to simply grasping the tool (Bryden et al., 2011). The present study thus sought to extend Bryden and Huszczynski’s (2011) protocol by establishing clear end goals with the intention to actually use the mug. More specifically, participants were asked to: (1) pick-up the mug, (2) pick-up the mug and pour a glass of water; (3) pick-up the mug and pass it to the confederate; and (4) pick-up the mug, pour a glass of water and pass it to the confederate. The inclusion of bimanual tasks in addition to unimanual tasks enabled us to further assess the role of task complexity. Many everyday activities require the two hands to act simultaneously when manipulating objects in the environment. Performance of bimanual actions requires a balance of intra- and inter-limb coordination (Bobbio et al., 2009); therefore more extensive cognitive processing is required (Logan and Fischman, 2011). It has been proposed that, when acting in bimanual tasks, the two hands play different, but complementary roles (Guiard, 1987; Haaland et al., 2004; Wang and Sainburg, 2007; Stone et al., 2013). According to the dynamic dominance hypothesis (e.g., Sainburg, 2002, 2005; Wang and Sainburg, 2007), the preferred hand acts to control limb dynamics (i.e., mobilizing), whereas the non-preferred hand is more adept at positional control (i.e., stabilizing). The current study sought to examine whether this division of labor extends to preferential reaching.

Also unique to the current study, is that participants were free to grasp the mug in whichever way they felt most appropriate. A mug can be grasped by the handle, through the handle, over the top, across the body, across the rim, etc. Tucker and Ellis (1998) demonstrated that viewing a handled mug is followed by reaching to grasp the handle for use. They used the term *micro-affordances* to describe that, within reaching space, vision of an object (i.e., location, shape, orientation) “will lead to activation of specific components of a reaching and grasping action” (Ellis and Tucker, 2000, p. 453). However, Lindemann et al. (2006) argued that activation only occurs when there is intention for use. As such, grasp postures have been classified as functional (i.e., grasp for use) and non-functional (i.e., unsuited for use; Randerath et al., 2009; Sunderland et al., 2011). Providing participants the freedom to grasp the mug without constraint in the current study enabled us to assess whether hand selection patterns observed by Bryden and Huszczynski’s (2011) were influenced by the requirement to grasp the handle, and enabled us to assess when participants freely chose to grasp the mug by the handle.

According to Rosenbaum’s concept of orders of planning for object manipulation (Rosenbaum et al., 2012), the way in which an individual grasps an object can be used to infer their mental state. From this perspective, first-order planning entails consideration of immediate task demands (i.e., reaching to grasp the mug). As an extension, second-order planning includes consideration of both the immediate and subsequent task. Beyond second-order planning, movements can be planned the *n*th order as a function of task complexity (see Rosenbaum et al., 2012 for a review). Less research has been devoted to higher order planning in independent object manipulation (e.g., Rosenbaum et al., 1990; Haggard, 1998); however, the manner in which objects are grasped and subsequently passed in joint action has become a topic of interest in recent years. For example, Gonzalez et al. (2011) had participants pick up and pass a tool (hammer, calculator, and stick) to a confederate, who would use it, or set it down. The initial orientation of the tool (facing toward or away) was manipulated to assess grasp postures. Results revealed participants demonstrated an initial grasp that fostered personal comfort and facilitated a comfortable and functional grasp posture for the confederate (Gonzalez et al., 2011). In line with Lindemann et al. (2006) and others, this was only observed when the confederate intended to use the object. Parallel observations have been reported in other joint action tasks (e.g., Ray and Welsh, 2011; Scharoun and Bryden, 2014), as such, the current study aimed to extend this work to a preferential reaching task.

Summarizing, the current study aimed to assess hand and grasp selection in both independent and joint action object manipulation within the context of a preferential reaching task. Mug location (right-space, midline, and left-space) and handle orientation (toward, away, to left, and to right) varied in four tasks that differed as a function of intention: (1) pick-up; (2) pick-up and pour; (3) pick-up and pass; and (4) pick-up, pour and pass. These tasks also differed as a function of the type of object manipulation (independent or joint action), hand requirements (unimanual or bimanual) and order of planning (first-order, second-order or third-order). Please refer to **Table 1** for a breakdown of task requirements. Dependent measures included the amount of preferred hand selection to pick-up the mug, and the proportion of trials where the mug was grasped by the handle. It was hypothesized that right-hand selection would increase as a function of object location (i.e., least in contralateral space and most in ipsilateral space) and complexity of the intended task (i.e., least in pick-up, most in pick-up, pour and pass). Furthermore, that the handle would be grasped in tasks which involved use (i.e., functional grasp: pick-up and pour, and pick-up, pour and pass).

**Table 1:** A breakdown of task requirements according to the type of object manipulation, hand requirements, and order of planning.

Task	Type of object manipulation	Hand requirements	Order of planning
Pick-up	Independent	Unimanual	First-order
Pick-up and pour	Independent	Bimanual	Second-order
Pick-up and pass	Joint action	Unimanual	Second-order
Pick-up, pour and pass	Joint action	Bimanual	Third-order


## Materials and Methods

### Participants

Thirty-nine right-handed undergraduate and graduate students (ages 18–30) from the university community participated in this study (see **Table 2**). Exact ages were not recorded. The Wilfrid Laurier University research ethics board approved all recruitment and testing procedures. Informed consent was obtained from all participants in accordance with the Declaration of Helsinki.

**Table 2:** Number of participants and mean (standard deviation) scores computed from the Waterloo Handedness Questionnaires.

	Total	Male	Female
*N*	39	14	25
WHQ	42.33 (10.61)	38.50 (14.43)	44.48 (7.20)


### Apparatus

#### Preferential Reaching Task

**FIGURE 1:**
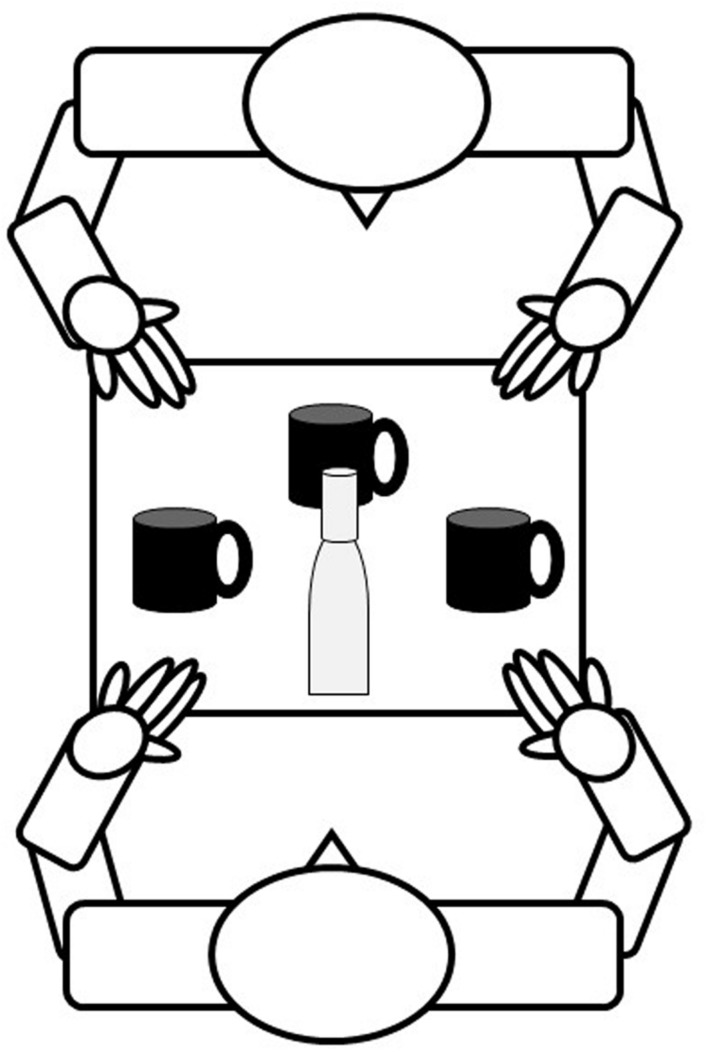
**Study set up.** Participant sat in the chair, researcher sat across from the participant.

Similar to Bryden and Huszczynski (2011), three mugs were placed in front of the participant in three regions of hemispace (left space, midline, and right space). The mug placed at the midline was positioned 90° in front of the participant, while mugs in left and right space were positioned at 0 and 180°. Mugs were located within reaching distance (approximately 30 cm from the midline) to afford a comfortable reach, while seated. Mugs were identical in size and shape; however, three different colors were included to allow the experimenter to refer to the mugs by color, as opposed to location. The mug handle was positioned toward the participant in a neutral position or to the left or right. Unique to this study, a fourth orientation (positioned away from the participant) was added. The set up also differed from Bryden and Huszczynski (2011) such that a water pitcher, with no handle, was placed in front of the participant at the midline (see **Figure 1**). Water was heated to make the pitcher warm to the touch, though not hot enough to cause discomfort or the risk of burning. This was done to mimic a warm beverage, such as tea or coffee. Also unique to this study, a researcher sat across from the participant and acted as a confederate in joint action tasks.

Participants were asked to: (1) pick-up the mug (i.e., pick-up); (2) pick-up the mug and pour a glass of water from the pitcher (i.e., pour); (3) pick-up the mug and pass it to a confederate (i.e., pass); and (4) pick-up the mug, pour a glass of water from the pitcher and pass it to a confederate (i.e., pour and pass). Participants began each trial with hands side-by side at the midline and were free to select the hand and grasp to complete the task. In other words, unlike Bryden and Huszczynski (2011), participants were allowed to grasp the mug with or without using the handle. There were 48 combinations of location, handle orientation, and task. Each combination was completed twice for a total of 96 trials. Trials were blocked by handle orientation; however, the order in which tasks appeared was randomized. A video camera was used to record participants’ hand movement behaviors. Videos were coded offline to record the percentage of right-hand (i.e., preferred hand) selection to pick-up the mug and the percentage of trials where the mug was grasped by the handle.

#### Waterloo Handedness Questionnaire (WHQ)

The 32-items Waterloo Handedness Questionnaire (WHQ; Steenhuis et al., 1990) was used to confirm participants’ hand preference based on self-report. The WHQ asks participants to indicate their preferred hand for 32 unimanual tasks. Each question permits five responses, where a number from -2 to +2 is used to compute a total handedness score from -64 to +64: *left always* (-2), *left usually* (-1), *both equally* (0), *right usually* (+1), and *right always* (+2). Left-handers are expected to show a negative score while right-handers are expected to show a positive score. No significant differences emerged in scoring between males and females [*t*(37) = -1.733, *p* = 0.091; see **Table 1**].

### Data Analyses

SPSS^©^ statistical software was used for data analyses. Each of the aforementioned dependent measures (right-hand selection, grasping mug by handle) from the preferential reaching task was submitted to a task (4: pick-up, pass, pour, pour and pass) by location (3: left space, midline, right space) by handle (3: right, left, toward, away) within-subject analysis of variance tests with repeated measures. It should be noted that the effects of sex were initially examined, but analysis revealed no significant differences in performance between males and females and therefore, will not be reported.

## Results

### Right-Hand Selection

Mauchley’s test of sphericity was violated for all within subjects’ effects. Huyn–Feldt corrections were applied when ε > 0.75 (Field, 2013). All other cases were corrected with the Greenhouse–Geisser estimate (Field, 1998). With the exception of the location factor and interaction between location and handle, Greenhouse–Geisser corrections were thus applied. It is important to highlight that, regardless of the correction, the same effects would hold.

**FIGURE 2:**
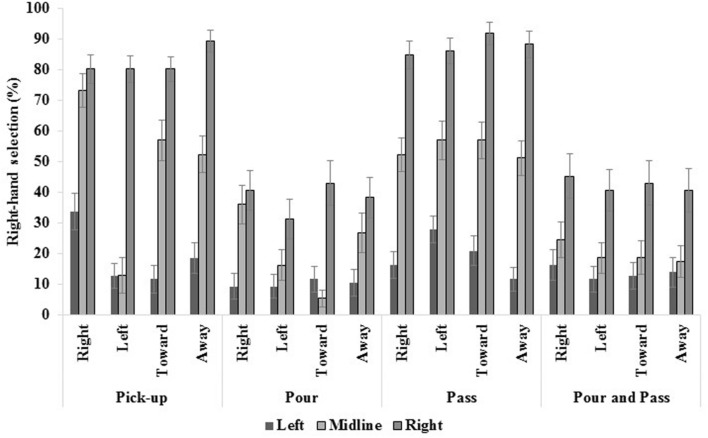
**The three-way interaction of task, location and handle revealed the most right-hand selection was displayed in right-space, among other effects, which are explained in text.** Standard errors bars are displayed.

Right-hand selection was significantly greater in right space (62.79%) compared to the midline (39.22%) and left space [15.55%; *F*(1.719,72.206) = 109.005, *p* < 0.001, η*^2^* = 0.722]. Furthermore, more right-hand selection was displayed in the unimanual tasks (pick-up = 54.56% and pass = 53.78%) compared to the bimanual tasks [pour = 23.22% and pour and pass = 25.29%; *F*(1.292,54.268) = 48.163, *p* < 0.001, η*^2^* = 0.534]. A main effect of handle [*F*(2.121,89.085) = 3.596, *p* = 0.029, η*^2^* = 0.079] also emerged; however, *post hoc* analyses using a Bonferroni correction for multiple comparisons revealed no differences in right-hand selection as a function of handle orientation. Significant two-way interactions are found in **Table 3**. These interactions are embedded within the significant three-way interaction of task, location and handle [*F*(10.519,441.787) = 2.874, *p* = 0.001, η*^2^* = 0.064; see **Figure 2**]; therefore, only the three-way interaction will be described in detail.

**Table 3:** Significant two-way interactions embedded within the three-way interaction of task, location, and handle.

Interaction	*F*-statement
Task × location	*F*(3.195,134.194) = 24.155, *p* < 0.001, η*^2^* = 0.365
Task × handle	*F*(5.797,243.484) = 3.845, *p* = 0.001, η*^2^* = 0.084
Location × handle	*F*(5.372,225.620) = 3.272, *p* = 0.006, η^2^ = 0.072


**FIGURE 3:**
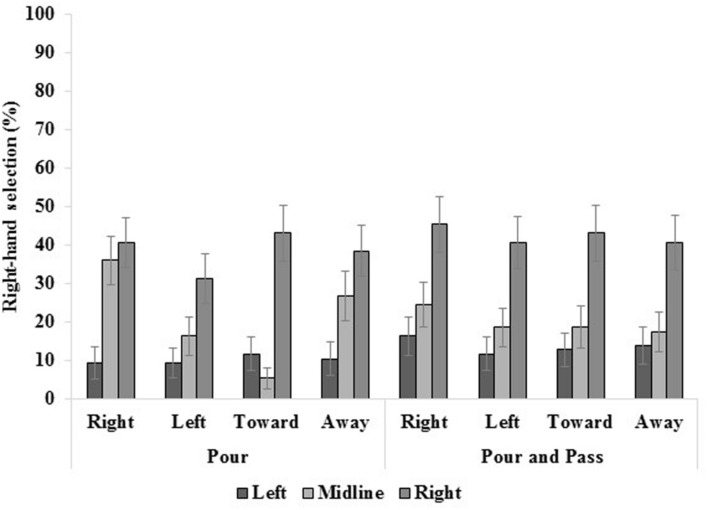
**The three-way interaction of task, location and handle, where only bimanual tasks are plotted (see supplementary material for additional data).** Although lower than what is normally observed in unimanual tasks, the most right-hand selection was displayed in right-space, among other effects, which are explained in text. Standard errors bars are displayed.

The aforementioned results are concurrent with previous reports with unimanual tasks (Bishop et al., 1996; Calvert, 1998; Mamolo et al., 2004, 2006; Bryden and Roy, 2006; Carlier et al., 2006). As such, remaining results will emphasize hand selection in bimanual tasks (pour, pour and pass – see **Figure 3**); however, detail concerning unimanual tasks (pick-up, and pass) can be found in the supplementary material.

In pour, when the mug was located in left space no differences emerged as a function of handle orientation. At the midline, the proportion of right-hand selection differed for all handle orientations. It was greatest when facing to the right, decreased when facing away from the participant and again when facing to the left. Right-hand selection was lowest when the handle faced toward the participant. Finally, when in right space, right-hand selection was greater when the handle faced toward the participant compared to the left; however, no other differences emerged. Also in pour, when the handle faced to the right and away from the participant, right-hand selection was greater in right space compared to the midline and left space, which also differed (i.e., more at midline versus left space). When facing to the left and toward the participant, greater right-hand selection was displayed in right space compared to both the midline and left space.

In pour and pass, no differences emerged as a function of handle orientation in left and right space. At the midline, right hand selection was greater when the handle faced to the right compared to away from the participant. Regardless of handle orientation, right-hand selection was greater in right space compared to the midline and left space, which did not differ.

### Grasping the Mug by the Handle

Mugs were grasped by the handle less often in passing (25.85%) compared to pick-up (53.21%), pour (51.23%) and pour and pass [42.74%; *F*(3,114) = 21.894, *p* < 0.001, η*^2^* = 0.366]. Furthermore, mugs were grasped less often by the handle when it faced away from the participant (21.15%) compared to the right (42.63%) or left (46.79%). The mug was grasped by the handle most frequently when it faced toward the participant [62.45%; *F*(3,114) = 36.419, *p* < 0.001, η*^2^* = 0.489].

Three separate two-way interactions emerged: (1) task by location; (2) task by handle; and (3) location by handle. Overall, mugs were grasped by the handle more frequently in independent tasks (pick-up; pick-up and pour) compared to joint action tasks (pass; pick-up, pour and pass). Furthermore, regardless of the mug’s location, the handle was grasped most often when it faced toward the participant and least often when it faced away. A more elaborate explanation of these interactions is provided in the follow.

**FIGURE 4:**
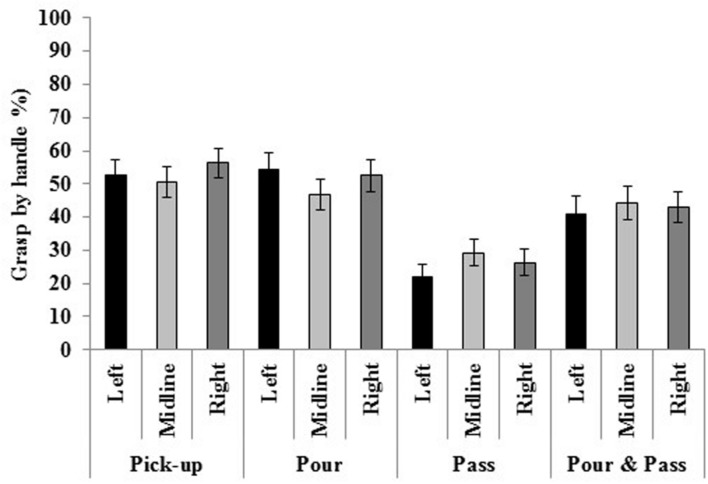
**Regardless of the handle orientation, participants grasped the mug by the handle most often in pick-up and pour.** Standard errors bars are displayed.

The significant interaction of task and location [*F*(6,228) = 3.670, *p* = 0.002, η*^2^* = 0.088; see **Figure 4**] identified that, in left space, mugs were grasped by the handle more often in pick-up and pour compared to pass and pour and pass. At the midline, mugs were grasped by the handle more frequently in pick-up, pour, and pour and pass compared to pass. In right-space, mugs were grasped by the handle most often in pick-up. Additionally, mugs were grasped by the handle more frequently in pour compared to pass, and pour and pass.

**FIGURE 5:**
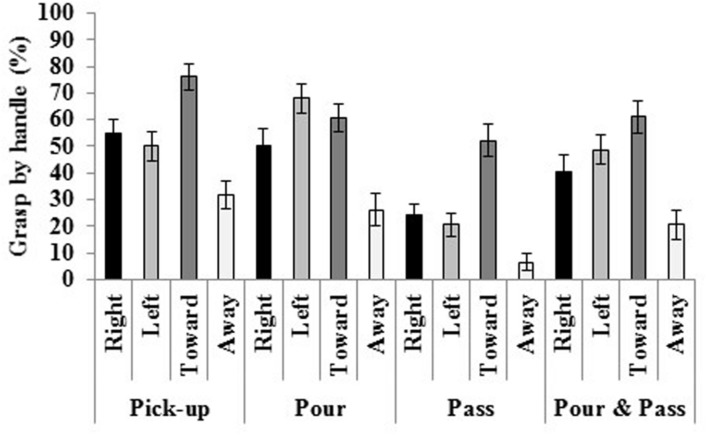
**The mug was grasped by the handle most often when it faced toward the participant, and when it faced to the left in pour.** Standard errors bars are displayed.

A task by handle interaction also emerged. Mauchley’s test of sphericity was violated [χ^2^(44) = 76.566, *p* = 0.002]; therefore a Greenhouse–Geisser correction was applied [*F*(5.959,226.447) = 7.997, *p* < 0.001, η*^2^* = 0.174; see **Figure 5**]. When the handle faced to the right, it was grasped more often in pick-up and pour compared to pass, and pour and pass. Furthermore, it was grasped more frequently in pour and pass versus pass. Facing to the left, the handle was grasped more often in pour compared to pick-up, pass, and pour and pass. Handles oriented toward the participants were grasped more frequently in pick-up compared to pour, pass, and pour and pass; whereas those facing away were grasped least often in pass compared to pick-up, pour, and pour and pass. In the pour task, the handle was grasped least often when facing away compared to all other tasks. In addition, the handle was more often when oriented to the left and toward the participant compared to when facing to the right and away. Finally, in pick-up, pass and pour and pass, the handle was grasped more frequently when facing toward and least often when facing away compared to all other tasks.

**FIGURE 6:**
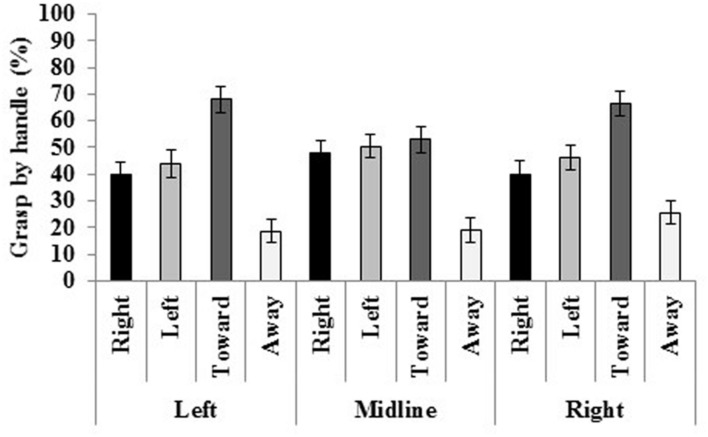
**Regardless of the mug’s location, the handle was grasped most often when it faced toward the participant and least often when it faced away.** Standard errors bars are displayed.

Finally, an interaction of location and handle was displayed. Mauchley’s test of sphericity was violated [χ^2^(20) = 44.281, *p* = 0.001]; therefore, a Greenhouse–Geisser correction was applied [*F*(4.747,180.381) = 8.135, *p* < 0.001, η*^2^* = 0.176; see **Figure 6**]. In both left and right space, the mug was grasped by the handle most often when facing toward and less often when facing away from the participants. Additionally, at the midline, it was grasped less frequently when facing away. When the mug’s handle was oriented to the right it was grasped more often at the midline. Facing left, it was also grasped more often at the midline, but only compared to left space. Oriented toward the participant, handles were grasped more often in left and right space compared to the midline. Finally when facing away, the mug was grasped by the handle most frequently in right space compared to the midline and left space.

## Discussion

Studies of hand selection in unimanual grasping report a preference for the right hand when picking up objects at the midline and in ipsilateral space, where right-handers are more likely to continue with this pattern of hand selection when reaching for objects in contralateral space (e.g., Bishop et al., 1996; Calvert, 1998; Mamolo et al., 2004, 2006; Bryden and Roy, 2006; Carlier et al., 2006). As evidenced in the interaction between task and location, findings from the current study provide additional support for this, such that right-hand selection was significantly greater in right space, and in unimanual tasks (pick-up, and pass).

In bimanual tasks (pour, and pour and pass), significantly less right-hand selection to grasp the mug was observed in comparison to unimanual tasks; however, the general pattern of greater right-hand selection in right space remained. Guiard (1987) was the first to suggest a tentative theoretical framework whereby the two limbs were seen to play distinct, but complementary roles when acting together. Regardless if the action was unimanual or bimanual, he suggested that each hand/hemisphere has a distinct role to play. More specifically, the right hand is essential for terminal accuracy, and the left hand for stabilization (Guiard and Ferrand, 1996). More recently, Sainburg and colleagues proposed the dynamic dominance hypothesis (e.g., Sainburg and Kalakanis, 2000; Sainburg, 2002, 2005; Przybyla et al., 2012), which indicates that the preferred hand is superior for precise control of movement and the non-preferred hand is more adept at positioning, as a result of hemispheric specialization (see Mutha et al., 2012 for a review). In the present work, participants demonstrated significantly less right-hand selection when grasping the mug, as it better afforded manipulating the pitcher; therefore, the left-hand was selected to stabilize the mug to receive the liquid.

Other recent work has provided support for complementary roles of the two hands when examining selection when performing bimanual skills. More specifically, Stone et al. (2013) had right-handed participants reach-to-grasp blocks scattered on a tabletop and subsequently construct 3D models. The use of both hands was required to perform the task successfully. Participants were free to build according to their comfort level (Experiment 1), on a large surface (Experiment 2) or a small surface (Experiment 3). Assessment of hand use identified that blocks were grasped with the right hand and stabilized with the left hand (Stone et al., 2013), similar to the current work which observed the pitcher grasped with the right hand and mug stabilized with the left.

Beyond a division of labor, differences in reaching behaviors have also been attributed to biomechanical constraints. As evidenced in the interaction between task and location, despite differences in hand selection in bimanual tasks, the general pattern of greater right-hand selection in right space remained. It can be argued that preferred hand use decreased in contralateral space to limit awkward grasping postures, similar to unimanual reaching. Therefore, in support of the *kinaesthetic hypothesis*, hand selection was also constrained by object proximity and efficiency of the movement (e.g., Gabbard and Helbig, 2004).

This finding is in contrast to Bryden and Huszczynski (2011), who noted that variance in selection patterns were attributed to the orientation of the mug’s handle. In their study, the greatest percentage of right-hand reaches occurred when the handle was oriented to the right, as expected. However, fewer right-hand reaches were observed when the handle was oriented to the left. Reaching to a left-oriented mug handle with the right-hand clearly requires an uncomfortable and inefficient movement; therefore, it was argued that object characteristics play an important role in limb selection (Bryden and Huszczynski, 2011). Unlike Bryden and Huszczynski (2011), participants in the present study were not required to grasp the mug by the handle. This was done to examine whether the foregoing hand selection patterns were influenced by the requirement to grasp the handle. In the current study, handle orientation did not influence right-hand selection; however, handle orientation did influence the tendency to grasp the mug by the handle. Specifically, the effect of handle orientation revealed that the handle was grasped least often when it faced away and most often when it faced toward the participant, where grasping behaviors were similar when the handle faced to the left and right. It can thus be argued that, when biomechanical constraints are strictly imposed by object characteristics (i.e., instructing participants to grasp the handle), this influences action selection. However, when participants are free to grasp an object (such as a mug) by any surface, this is not the case, as hand and grasp selection are adjusted to maximize biomechanical efficiency. In this context, and similar to Gabbard and Helbig (2004), the influence of object proximity on hand selection remains.

Tucker and Ellis (1998) reported that, when one views a handle mug, reaching to grasp the handle is a typical response (Tucker and Ellis, 1998). However, it has also been argued this is only the case when an individual intends to use the object (Lindemann et al., 2006). Results of the current study indicate that the intended action does indeed influence the decision to grasp a mug by the handle; however, not to the same extent as previously reported. Here, as displayed in a main effect of task, the handle was grasped more often in independent manipulation (i.e., pick-up, pour) than in joint action (pass, pour and pass). Furthermore, when joint action required independent manipulation prior to passing (i.e., pour and pass), the mug was grasped more by the handle. Summarizing, participants executed a functional grasp (i.e., grasping by the handle) more often when manipulating the mug independently. Interestingly, this did not differ when comparing first-order (pick-up) to second order (pour) planning (Rosenbaum et al., 2012). This finding is in contrast to Lindemann et al. (2006), as no clear distinction between grasping and grasping for use was observed.

Also evidenced in the main effect of task, there was a greater tendency for participants to grasp the mug by the handle when the participant had to pour prior to passing (third-order planning), compared to when the participant was simply required to pick-up and pass the mug (second-order planning). Differences in action intention have been previously shown to alter movement behavior in joint action (see Becchio et al., 2010 for a review). Distinct kinematic patterns have been observed for cooperation and competition in comparison to individual movement patterns. More specifically, cooperative tasks reveal a strong relationship between the two participants’ kinematics, unlike the competitive task (Georgiou et al., 2007). When participants are instructed to cooperate, yet the confederate acts competitively, the participant’s kinematics also reflects a more competitive pattern (Becchio et al., 2008). Additional evidence is derived from doorway holding. Santamaria and Rosenbaum (2011) observed that a person who opens a door will continue to hold the door for a follower if he/she expects a shared belief that the combined total effort is less than the sum of individual efforts. Furthermore, the follower will speed up to decrease the door holding time (Santamaria and Rosenbaum, 2011). Interpreting findings from the current study in light of the aforementioned literature, it can be argued that grasp selection reflects consideration of the confederate’s initial grasp behavior (e.g., Gonzalez et al., 2011; Ray and Welsh, 2011; Scharoun and Bryden, 2014); however, this is ultimately limited by the participants’ actions. When passing an object, individuals are cognizant of the recipient’s initial grasp posture. Nevertheless, if a skilled action (i.e., pouring water into a mug) is required prior to passing, the actor is likely to prioritize their own grasp posture over that of a confederate. This is likely a consideration of shared-effort (Santamaria and Rosenbaum, 2011).

Summarizing, the current study provides additional support for a right hand preference in unimanual grasping. Furthermore, this study extends existing literature that has identified role differentiation between the two hands, to a preferential reaching task. Finally, the current work provides support for an increase of right hand selection in right space, regardless if the task is unimanual or bimanual in nature. This is likely attributed to the propensity to maximize biomechanical efficiency with hand selection. Continuing with the notion of biomechanics, in reference to grasp selection and the propensity for participants to grasp the mug by the handle, unlike previous findings that noted imposed constraints (i.e., instruction participants to grasp the handle; Bryden and Huszczynski, 2011) influence action selection, when afforded the freedom to grasp a mug by anysurface, grasp postures are adjusted to maximize efficiency. This is not limited to independent manipulation, but also extends to joint action. Here, grasp selection reflects offering a confederate a functional grasp (i.e., grasping by the handle) only if the intended action of the confederate is similar or costs less effort than that of the participant. Together, findings from the current study add to our knowledge of hand and grasp selection in unimanual and bimanual object manipulation, within the context of both independent and joint action tasks. It is argued that early unimanual hand use may linked to later role-differentiated bimanual manipulation (e.g., Nelson et al., 2013); therefore, this study provides a foundation for investigating unimanual and bimanual hand selection from a developmental perspective. This research is currently underway in our lab.

## Author Contributions

KS completed a portion of the study for her 4th year thesis, supervised by Dr. PB. SS has collected additional data and wrote the majority of the paper, under the guidance and supervision of Dr. PB.

## Conflict of Interest Statement

The authors declare that the research was conducted in the absence of any commercial or financial relationships that could be construed as a potential conflict of interest.
