# Supplementary material for: Hand and Grasp Selection in a Preferential Reaching Task: The Effects of Object Location, Orientation, and Task Intention
Source: Front Psychol. 2016 Mar 16;7:360. doi: 10.3389/fpsyg.2016.00360 (PMC4793775; doi:10.3389/fpsyg.2016.00360)
Supplement: Supplementary file 1 [file Data_Sheet_1.DOCX]

**Supplementary Material**

**Right-hand selection**.

In pick-up, when the mug was located in left space, right-hand selection was greater when the handle was oriented to the right, compared to all other orientations. At the midline, there was no difference when the handle was oriented to the right or left; however, right-hand selection was greater with these two handle orientations compared to when facing towards or away from the participant. Finally, in right space, greater right-hand selection was displayed when the handle faced away from the participant compared to toward; however, no other differences emerged. Next, in pour, when the mug was located in left space no differences emerged as a function of handle orientation. At the midline, the proportion of right-hand selection differed for all handle orientations. It was greatest when facing to the right, decreased when facing away from the participant and again when facing to the left. Right-hand selection was lowest when the handle faced toward the participant. Finally, when in right space, right-hand selection was greater when the handle faced towards the participant compared to the left; however, no other differences emerged. In the pass task, when the mug was located in left space, right-hand selection was greater when the handle faced to the left compared to the right and away from the participant. No other differences emerged. Finally, in pour and pass, no differences emerged as a function of handle orientation in left and right space. At the midline, right hand selection was greater when the handle faced to the right compared to away from the participant.

In pick-up, when the handle faced to the right, right-hand selection was greater at the midline and in right space compared to left space. When facing to the left, towards and away from the participant, right-hand selection was greater in right space compared to both the midline and left space, where right-hand selection at the midline was greater than in left space. In pour, when the handle faced to the right and away from the participant, right-hand selection was greater in right space compared to the midline and left space, which also differed (i.e., more at midline vs. left space). When facing to the left and toward the participant, greater right-hand selection was displayed in right space compared to both the midline and left space. In pass, regardless of handle orientation, right-hand selection was greatest in right space compared to the midline and left space. Furthermore, significantly greater right-hand selection was displayed at the midline compared to left space. Finally in pour and pass, regardless of handle orientation, right-hand selection was greater in right space compared to the midline and left space, which did not differ.

In left space, when the handle faced to the right, right-hand selection was greater in pick-up compared to all other tasks. Additionally, there was greater right-hand selection in pass compared to pour. When the handle faced to the left, greater right-hand selection was demonstrated in pass compared to all other tasks. No other differences emerged. At the midline, when the handle faced to the right, the proportion of right-hand selection differed in all tasks. It was greatest in pick-up, then pass, then pour and finally pour and pass. When the handle faced to the left and away from the participant, there was no difference in pick-up and pass; however, right-hand selection was significantly greater in these tasks compared to pour and pour and pass. Finally, in conditions where the handle faced toward the participant, right-hand selection was greatest in pick-up and pass, which did not differ. Significantly less right-hand selection was displayed in pour and pass. The least right-hand selection was displayed in pour. Finally, in right space, in conditions where the handle faced to the right, and away from the participant, the most right-hand selection was displayed in pick-up and pass, which did not differ, and the least was demonstrated in pour and pour and pass, which also did not differ. When facing to the left, the proportion of right-hand selection followed a similar pattern; however, there was significantly less right-hand selection in pour compared to pour and pass. Finally, when facing toward the participant, the most right-hand selection was displayed in pass compared to all other tasks, where pick-up had the next greatest proportion. The least right-hand selection was displayed in pour and pour and pass, which did not differ.
